# Causal inference and observational data

**DOI:** 10.1186/s12874-023-02058-5

**Published:** 2023-10-11

**Authors:** Ivan Olier, Yiqiang Zhan, Xiaoyu Liang, Victor Volovici

**Affiliations:** 1 Data Science Research Centre, Liverpool John Moores University, Liverpool, UK; 2 Institute of Environmental Medicine, Karolinska Institutet, Stockholm, Sweden; 3 Department of Epidemiology and Biostatistics, Michigan State University College of Human Medicine, East Lansing, MI 48824 USA; 4 Department of Neurosurgery, Center for Medical Decision Making, Erasmus MC, Rotterdam, The Netherlands

## Abstract

Observational studies using causal inference frameworks can provide a feasible alternative to randomized controlled trials. Advances in statistics, machine learning, and access to big data facilitate unraveling complex causal relationships from observational data across healthcare, social sciences, and other fields. However, challenges like evaluating models and bias amplification remain.

## Main text

Billions of data records are generated every day, facilitating the discovery of knowledge. Particularly, medical, epidemiological, and social science research has significantly benefited from the vast amount of data available through sources such as medical records, easily attainable surveys, and social media platforms. This availability has led to a significant increase in the popularity of observational studies and meta-analyses as complementary approaches of randomized controlled trials (RCTs). RCTs are considered as the gold-standard study design for decision-making. However, conducting RCTs may not always be feasible due to ethical concerns, significant costs, or time limitations. Traditionally, outcomes from observational studies are considered of less value than RCTs, mainly because the former are vulnerable to confounding and bias issues. Recently, novel developments in statistics and machine learning (ML) are driven the development of causal inference in observational studies to the point of serving as a feasible substitute or complement for RCTs in decision-making [1]. Most statistical and ML methods are designed to establish an association map between input (factors) and output (target) variables. However, such association maps are unable to identify potential latent factors that influence both inputs and outputs, making their use limited to determining causal links. For instance, several studies reported a higher prevalence of lung cancer among coffee drinkers compared to non-drinkers. However, since many coffee drinkers also smoke, the observed association between coffee drinking and lung cancer is confounded by smoking, the true cause of the disease [2].

Causal inference from observational data finds application across various fields, with notable impact observed in domains such as healthcare, medicine, political and economic sciences, and social sciences. In healthcare and medical research, causal inference enables the identification of heterogeneous treatment effects and the formulation of personalized treatment strategies. By incorporating individual-level data, genetic information, and ML techniques, the field of personalized medicine benefits from enhanced causal inference methodologies [3]. The critical role of causal inference extends to policy evaluation and intervention assessment, where advancements in causal inference methods facilitate evidence-based decision-making by rigorously evaluating policy effectiveness, estimating causal impacts, and comprehending unintended consequences. Additionally, the utilization of instrumental variables, regression discontinuity designs, and quasi-experimental approaches as methodological advancements further augment the understanding of complex social phenomena, policy impacts, and economic relationships [4, 5].

Broadly speaking, causal inference attempts to build data-driven models that can predict the effect of interventions on outcomes. Using observational data for causal inference is gaining momentum due to the confluence of factors such as the large amount of more complex and richer data and advanced techniques from statistics and ML. In general, two frameworks exist for causal inference in observational studies, which are not necessarily mutually exclusive: the structural causal model (SCM) framework and the potential outcome framework (POF). The SCM framework relies on deterministic, functional equations to construct directed acyclic graphs (DAGs) with variables as nodes and links as causal relationships and is particularly useful in identifying unknown causal and confounding variables while estimating the actual effect of a given treatment. On the other hand, the POF framework (also known as the counterfactual framework) examines outcomes that would have likely been observed had the treatment differed, representing the counterfactual or the missing outcome. Other frameworks such as instrumental variables, mediation analysis, and Bayesian networks are also noteworthy in causal inference research [6].

In recent years, there has been growing interest in combining multiple frameworks and approaches to improve causal inference. Integrating ideas from different frameworks can lead to more comprehensive and robust causal analyses. Additionally, the use of machine learning techniques and the exploration of new identification strategies are areas that hold promise for advancing causal inference research [7]. Analysis of observational studies could benefit from the best of two worlds. ML methods can help identify confounding variables, handle high-dimensional data, and improve prediction accuracy, while causal inference provides interpretability and causal understanding. Integrating these fields can lead to more powerful and robust causal inference models [8].

Causal inference research is a dynamic field that continues to evolve. Numerous real-world scenarios entail complex systems comprising multiple interacting variables. Advances in causal inference are instrumental in unraveling causal relationships in such systems. The availability of large-scale datasets presents both opportunities and challenges for causal inference. The development of scalable methods capable of efficiently handling large data sets while addressing biases, confounding, and selection effects constitute an active area of research. Furthermore, efforts are being made to devise methodologies for extracting causal relationships from unstructured data and integrating them with structured data, thereby enhancing the depth of insights and broadening the applicability of causal inference from observational data.

However, causal inference with observational data is not free of challenges. For instance, causal inference models are hard to evaluate. If a causal link is found, still there is no clear mechanism to assess whether the link is real or not. The performance of associative data-driven models can be assessed and compared easily since large data repositories are publicly available and widely used. However, this is not the case for causal inference, for which the lack of public benchmark data is one of the biggest problems it is encountered in their development. There is also a lack of comparisons to non-causal methods in the literature [9]. It is also inevitable to make untestable assumptions, which could also contribute to bias amplification and harm the external validity when compared to non-causal counterparts [10].

As the field continues to advance, interdisciplinary collaborations, methodological innovations, and the integration of emerging technologies will continue to expand the frontiers of causal inference and its applications in various domains. Nevertheless, challenges must be addressed for swift adoption in social and medical research.

## Data Availability

Not applicable.

## Abbreviations

DAG: directed acyclic graphs
ML: machine learning
POF: potential outcome framework
RCT: randomized control trial
SCM: structural causal model
